# Sound Reception in the Yangtze Finless Porpoise and Its Extension to a Biomimetic Receptor

**DOI:** 10.3390/biomimetics8040366

**Published:** 2023-08-15

**Authors:** Zhongchang Song, Wenzhan Ou, Jiao Li, Chuang Zhang, Weijie Fu, Wenjie Xiang, Ding Wang, Kexiong Wang, Yu Zhang

**Affiliations:** 1 Key Laboratory of Underwater Acoustic Communication and Marine Information Technology of the Ministry of Education, Xiamen University, Xiamen 361005, China; songzhongchang@foxmail.com (Z.S.); chuangzhang@stu.xmu.edu.cn (C.Z.); 22320220156419@stu.xmu.edu.cn (W.F.);; 2 State Key Laboratory of Marine Environmental Science, College of Ocean and Earth Sciences, Xiamen University, Xiamen 361005, China; 3 Institute of Hydrobiology, Chinese Academy of Sciences, Wuhan 430072, China

**Keywords:** finless porpoise, directional reception, acoustic structures, numerical simulation

## Abstract

Sound reception was investigated in the Yangtze finless porpoise (*Neophocaena phocaenoides asiaeorientalis*) at its most sensitive frequency. The computed tomography scanning, sound speed, and density results were used to develop a three-dimensional numerical model of the porpoise sound-reception system. The acoustic fields showed that sounds can reach the ear complexes from various pathways, with distinct receptivity peaks on the forward, left, and right sides. Reception peaks were identified on the ipsilateral sides of the respective ears and found on the opposite side of the ear complexes. These opposite maxima corresponded to subsidiary hearing pathways in the whole head, especially the lower head, suggesting the complexity of the sound-reception mechanism in the porpoise. The main and subsidiary sound-reception pathways likely render the whole head a spatial receptor. The low-speed and -density mandibular fats, compared to other acoustic structures, are significant energy enhancers for strengthening forward sound reception. Based on the porpoise reception model, a biomimetic receptor was developed to achieve directional reception, and in parallel to the mandibular fats, the silicon material of low speed and density can significantly improve forward reception. This bioinspired and biomimetic model can bridge the gap between animal sonar and artificial sound control systems, which presents potential to be exploited in manmade sonar.

## 1. Introduction

The biosonar system of odontocetes has excellent target localization and discrimination abilities in aqueous environments, which has been the subject of numerous studies [1,2]. Their biosonar has been quoted as outperforming current manmade sonar systems in many aspects [3], including sound transmission, sound reception, and target detection [4,5,6,7]. Odontocetes generate and transmit sounds in the forehead. As sounds propagate forward into the forehead, the acoustic structures, including the melon, dense connective tissue layer, upper jaw, nasal passages, and air sacs, modulate sounds into a narrow, forward-oriented beam [8,9]. Beam formation involves a series of stages [10], and the respective functions of various anatomical structures involved in beam modulation have been investigated through modeling [11,12,13,14], suggesting skull structures and acoustic fats are important in achieving directional transmission.

Acoustic fats in the mandibular region and mandible are key to receiving sounds in many pathways [5,15,16,17,18]. The widely accepted reception pathways are composed of the mandible and mandibular fats. Norris proposed that sounds enter the reception system through the low-sound-speed external mandibular fat and then traverse the caudal portion of the mandible to reach the internal mandibular fat before finally arriving at the bony complexes [19,20]. This “jaw hearing” pathway was widely accepted and supported in psychoacoustic studies and numerical simulation [5,21]. The knowledge of sound reception in odontocetes has been expanded since the new century. The three-dimensional modeling on the hearing of the common dolphin (*Delphinus delphis*) indicated that mandibular fats can facilitate sound reception from forward directions [5]. Cranford et al. developed a numerical model of a Cuvier’s beaked whale (*Ziphius cavirostris*) and found an additional sound entrance through the ventral margin of the mandible [15]. The solid mandible was thought to be an important guide to conduct sounds to the bony complexes as well, as demonstrated in auditory examinations on beluga whales (*Delphinapterus leucas*), indicating a high sensitivity to sound stimulation from the rostrum tip [22,23]. This location might also be the entrance of the effective acoustic channels for the Risso’s dolphin (*Grampus griseus*) and the Yangtze finless porpoise (*Neophocaena phocaenoides asiaeorientalis*) [24,25]. Song et al. found that the front portion of the mandible was the entrance of an alternative reception pathway for the finless porpoise (*Neophocaena asiaorientalis sunameri*), which might be associated with the high-sensitivity areas found at the rostrum tips of Beluga whales, Risso’s dolphins, and Yangtze finless porpoises [17]. The mandible tip was found to induce elastic waves propagating along the mandible posteriorly to merge with those from the mandibular fats.

This bone conduction phenomenon was observed in a later study on a short-beaked common dolphin skull [18]. The elastic waves in the mandibles were informative to localize source positions via binaural cues. The skull vibration, though not examined in detail, may have already had some input to the measured elastic waves [26]. The role of the mandible may become negligible in other pathways; Cranford et al. found that in the “gular pathway”, as sounds propagate to the posterior part of the mandible, it becomes increasingly transparent posteriorly. In comparison, the soft tissue and the internal mandibular fat body complexes played the main roles in conducting sounds to the ear bony ear complex [15].

These aggregations showed the complexity of acoustic windows in odontocetes. Though the most sensitive areas (the positions of acoustic windows) may have been demonstrated in many species [22,23,24,25,27], there might be other sound-reception paths that have not been addressed in detail as well as the roles of the various structures in these paths. The head as a whole may be considered a volume antenna [15,27], and thus, the roles of the various structures, including the mandible, the mandibular fats, soft tissue, and skull, in conducting sounds to the ear complex require additional work to elucidate. In this study, we extended the research to investigate sound reception in the Yangtze finless porpoise, suggesting the complexity of sound reception in this species, and herein, we discuss the influences of the low-speed and -density acoustic fats on receptivity.

The information we obtained concerning the porpoises’ reception is promising to initiate biomimetic research. Investigations into the directional sound transmission and reception pathways in odontocetes can help to inspire biomimetic ideas on system design to obtain extra gains in underwater target detection [28]. Actually, artificial designs inspired by odontocetes biosonar have been proposed and physically built to facilitate controls in sound transmission and reception [29,30,31], suggesting the reliability of transition from basic bioacoustics research to bioinspired applications. Regarding practical applications, additional information is needed to find ways of incorporating the inner workings of odontocete biosonar into manmade sonar systems. This paper was extended from our previous studies [32,33] and provided additional information on the sound reception of the species. Here, we address the roles of acoustic structures in directional sound reception in the Yangtze finless porpoise. More importantly, a biomimetic model was proposed, and its directional sound reception was investigated. The results are meaningful to strengthen our understanding of sound reception in this species and provide a reference for biomimetic research.

## 2. Materials and Methods

### 2.1. Computed Tomography Scanning and Reconstruction

**Figure 1:**
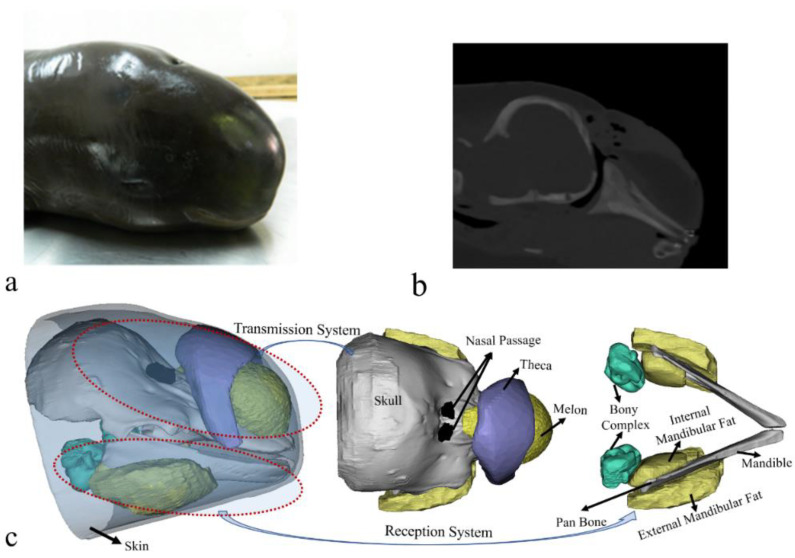
(**a**) A photo of the finless porpoise. (**b**) A computed tomography scanning image of the porpoise head in a sagittal cross-section. (**c**) Reconstruction of the finless porpoise showing the forehead transmission system and reception system in the lower head. The transmission system in the forehead includes the cranium, nasal passage, theca, melon, and upper jaw components. The reception system in the mandibular region consists of ear bones (bony ear complex), internal mandibular fat, external mandibular fat, and mandible.

We opportunistically took advantage of a stranded and dead Yangtze finless porpoise to reconstruct its biosonar system. CT scanning was conducted on the specimen the day after its death. A SOMATOM Definition Dual Source CT Scanner (DSCT; Siemens, Munich, Germany) with a resolution of 1 mm was used in the Radiology Department of Zhongshan Hospital of Wuhan University (Wuhan, China). The images were collected at a power setting of 120 kV × 76 mA and saved in IMA format for subsequent analysis. The biosonar system was reconstructed in three dimensions (Figure 1). More details concerning the procedures to reconstruct the sound speed and density can be found in our previous paper [32]. The sound-transmission system in the forehead, represented by the theca (a region of connective tissue overlying the caudal portion of the melon), melon, nasal passage, and skull structures, including the cranium, can be clearly visualized (Figure 1). The anatomical arrangement of these structures was similar to that reported in other odontocetes [17,34]. The structures comprising the sound-reception system in the lower jaw region were also visualized, including the mandible, acoustic fats (external and internal mandibular fat bodies) encasing the mandible, and bony complexes adjacent to the internal mandibular fat bodies. The fine reconstructions made plausible the subsequent model development.

### 2.2. Numerical Modeling and Computation

**Figure 2:**
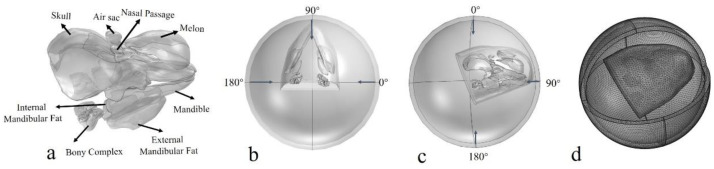
(**a**) The geometry model of the biosonar reception system, including the skull, bony ear complex, internal mandibular fat, external mandibular fat, air component (air sac and nasal passage), melon, and mandible. (**b**) The incident background field in the horizontal plane, where arrows depict the incident angles. (**c**) The incident background field in the vertical plane. (**d**) The numerical model of the porpoise head and computing domain after meshing.

To investigate the directional sound reception, sounds were set to be incident from different angles and the background incidence field as follows:

(1) $$p_{b}=p_{0}\frac{r_{re}}{r_{s}}e^{-ikr_{s}}$$

where *r_re_* = 1 m, *r_S_* = |*x* − *x*_0_|, *k* is the wave number, *x* denotes the location of the spherical wave, and *x*_0_ is the location on the boundary. *p_b_* and *p*_0_ represent the pressure at *x* and *x*_0_, respectively. The first set of simulations was presented by introducing *φ* and *θ* to represent incidence angles from horizontal and vertical planes, with a step of 3° for each plane, both ranging from 0° to 180° (Figure 2). The initial incident pressure was set as 1 Pa. The absolute pressures on the surface of the left and right bony complexes were integrated to compute the monaural directivity for each ear complex and then added to create a binaural directivity.

The directivities of sound reception were addressed for 54 kHz at frequency domain, at which the finless porpoise had the best hearing sensitivity [35,36]. The COMSOL Multiphysics software (Stockholm, Sweden) was used to solve the volume deformations in fluid media and shear deformations in solid media. The whole computing domain was meshed into elements one-fifth of the wavelength. The boundary conditions were set between different media. In fluid media, including mandibular fats, melon, air, water, and other soft tissues, the sound pressure and normal velocity at the contact boundary of different media were set as continuous (that is, *p*_1_ = *p*_2_ and *v*_1_ = *v*_2_). For the boundary between solid mandible/skull and fluid media, the normal velocity and mechanical stress were continuous (*v*_1_ = *v*_2_ and *T*_1_ = *T*_2_).

**Figure 3:**
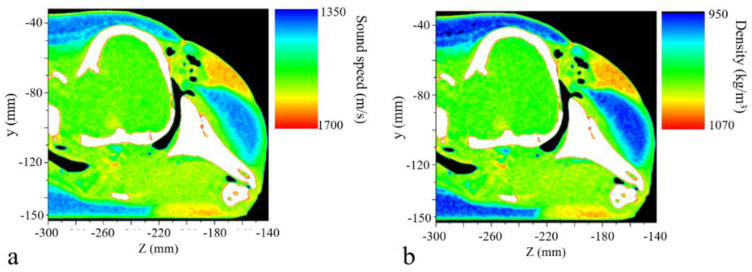
The reconstructed (**a**) sound speed and (**b**) density distributions of the porpoise’s head at a sagittal plane.

We assigned 1500 m/s and 998 kg/m^3^ as the sound speed and density of water, respectively. These values were 343 m/s and 1.21 kg/m^3^ for the air-filled nasal passage and sacs. For soft tissues, the settings were set according to the reconstructions of sound speed and density of the porpoise (Figure 3). The acoustic impedance of the soft tissues in the mandibular region was close to that of water, providing a fine impedance matching and low-energy-loss channel for sounds to enter the odontocete head [32,37,38]. More details concerning computed tomography scanning, sound speed, and density measurements can be found in our previous paper [32]. The compressional and shear wave speeds of the skull structures were set as 3380 m/s and 2200 m/s respectively, and the density was 2035 kg/m^3^ [16,39].

The numerical model of the species is verified in our previous paper [32], suggesting that the simulated signals in the far field had similar temporal and spectral characteristics to those recorded from real porpoise [40]. Thus, we directly used the numerical modeling and set six cases to address the influence of the various structures on sound-reception directivity. A full set of acoustic structures, including mandible, mandibular fats, melon, skull, and air sac, was considered as case I. In the rest of the cases, the acoustic structures were replaced by water, making different combinations. The skull, melon, mandibular fats, air components, and mandible were individually removed from case I, creating case II (No_Skull), case III (No_Melon), case IV (No_Fat), case V (No_Air), and case VI (No_Mandible) correspondingly.

### 2.3. Biomimetic Receptor

**Figure 4:**
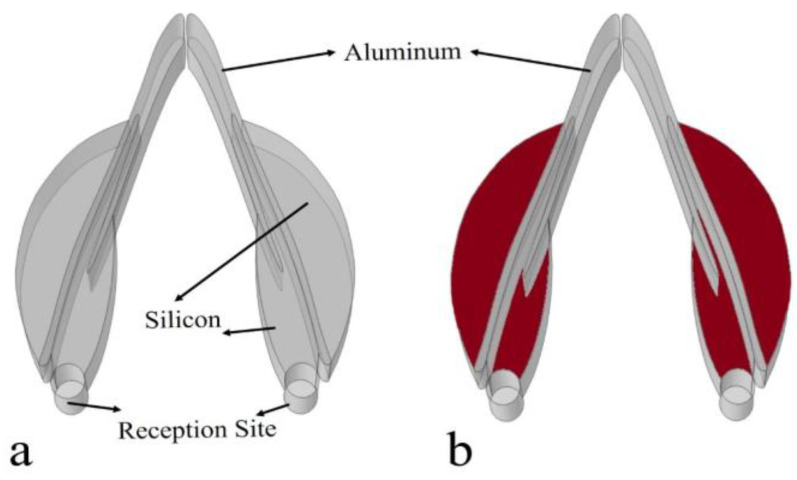
(**a**) A biomimetic sound receptor, where aluminum (solid) and silicon were used as composites of the mandible and internal and external mandibular fats, respectively. (**b**) The biomimetic model in which the low-speed silicon materials (red) were replaced by water.

The Yangtze River finless porpoise’s reception system was not symmetrical concerning anatomy and the sizes of the respective structures (Figure 2a). We referred to the animal’s reception system but simplified the biomimetic model as a symmetrical system, termed as the bio-receptor (Figure 4a). The mandible was replaced by aluminum (shear wave speed: 3080 m/s; longitudinal wave speed: 6260 m/s; density: 2700 kg/m^3^), and both the external and internal mandibular fat bodies were mimicked using silicon, with a sound speed of 1050 m/s and density of 1050 kg/m^3^. The directional reception was first examined for the biomimetic model by using the background incidence field setting as well. The directivity was examined at 54 kHz. Furthermore, another biomimetic model was developed in which the silicon was replaced by water (Figure 4b). These two models were compared to emphasize the variability of directional reception.

## 3. Results

**Figure 5:**
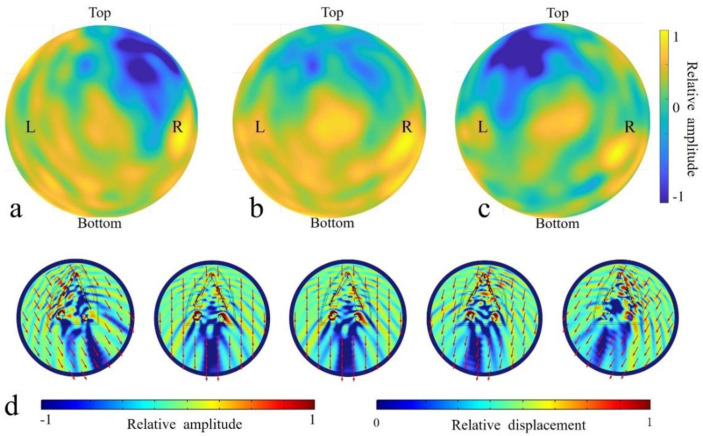
The simulated receptivity for 54 kHz sources at the inner ears of the full model. (**a**) The monaural directivity for the left ear complex. (**b**) The binaural directivity estimated using two monaural ear complexes. (**c**) The monaural directivity for the right ear complex. (**d**) The acoustic fields on the head surface resulting from incidence from 120°, 92°, 90°, 78°, and 60° (from left to right) in the horizontal plane for 54 kHz.

The monaural reception directivity of the left ear complex and right ear complex and binaural directivity of the two ear complexes were determined for sounds of 54 kHz (Figure 5). Sounds incident from left side resulted in many receptivity peaks, showing a relatively spreading spatial distribution. Several peaks were found on the same side, and a local peak was surprisingly found in the opposite ear. Comparably, sounds received at the right ear complex had a distinct maximum from the right incidence angles. A shadowed region (depicted in blue) was found in the top-right area for the left ear complex, and the shadowed area shifted to the top left for the right ear complex. The binaural receptivity (Figure 5b) formed from the combination of the left and right monaural directivities demonstrated that the reception was enhanced in the forward direction. The right ear peaks were raised in the bottom-right region. The reception patterns clearly showed various peaks, including left ear, right ear, and forward peaks. Local maxima were seen in an extensive spatial scale below the forward horizon.

To better illustrate the internal reception details, acoustic fields were illustrated in the horizontal (Figure 5d) plane for the full model (case I). Apparently, the head shielded the sound waves in the upper head, resulting in amplitude differences on the top of the head. The differences between the front and back head showed that there was a shadowed area regardless of the incident angle. The location of the shadowed areas corresponded to the incidence angle. For sounds incident from the right-forward direction (90°), the shadow area was quasi-on-axis, and the rest shadowed areas were unilateral, with the low acoustic fields distributed consistently on the other side of body axis. Sounds originating from the animals led to vibrations in the mandible and sound wave propagation in the mandibular fats. Sounds incident from the 60° (right side) corresponded to a stronger mandible vibration at the same side (Figure 5d), especially at the anterior portion. And this trend was reversed for sounds incident from 120° (left side), in which case the left mandible had a much stronger vibration amplitude anteriorly. The vibrations at the ear complexes were the lowest when stimulated by sounds from 60° in the horizontal plane.

Apparently, the head was a shield, and a strong shadowed area formed for all incident angles. The presences of the mandible and mandibular fats formed a waveguide for sounds to propagate to the ear complexes. Sounds from the forward directions stimulated the mandible tip to vibrate, and then, the waves were partially transmitted to the internal mandibular fat before eventually arriving at ear complexes. This bone conduction pathway was verified again as consistent with our previous simulation [17].

**Figure 6:**
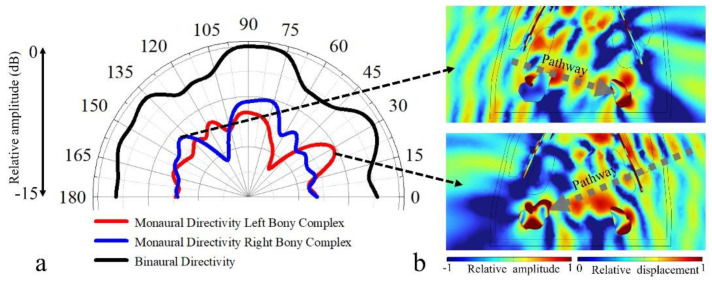
(**a**) The binaural and monaural sound-reception directivities estimated at 54 kHz in a chosen horizontal plane. The monaural directivity was estimated using sound pressure received in surfaces of left hearing bone and right hearing bone, while the “binaural” case means the normalized distributions from left and right hearing bones are summed. (**b**) Acoustic field details of sounds incident from 141° (top) and 30° (bottom) in the horizontal plane, where sound from the left side (141°) stimulated strong vibrations at the right ear complex through a subsidiary pathway, and sounds from the right side (30°) stimulated strong vibrations at the left ear complex.

The receptivity was depicted for the chosen horizontal plane with incident angles ranging from 0° to 180° (Figure 6). The monaural directivities showed that sounds incident from the ipsilateral side of each ear complex stimulated strong surface pressure at the complex locally from 60° to 120°. Moreover, we identified a receptivity peak at the other side of the reference axis for each ear complex (90°). After merging the sound pressure integrals of both ear complexes to acquire a binaural directivity, we found that sounds incident from forward directions had a higher reception efficiency than off-axis stimulations, and the −3 dB beam width was 62° (60° to 122°) in the horizontal plane.

Sounds incident from the 30° (right side) showed an effective propagation to the left ear complex as well, and those incident from 140° (left side) led to a receptivity peak at right ear complex. These lateral maxima on the sides opposite each ear may correspond to subsidiary hearing pathways (Figure 6b). The multiple maxima identified in the receptivity patterns demonstrated the complexity of sound reception in the finless porpoise, and the whole head, especially the lower head, may function in receiving sounds [5].

**Figure 7:**
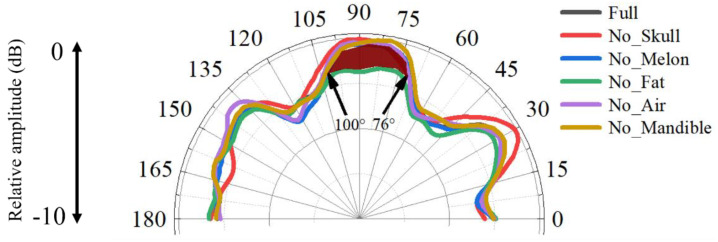
The sound-reception directivities examined in the horizontal plane for the full model and the models without skull, without mandible, without melon, without mandibular fats, and without air at 54 kHz.

We further extended the simulations to address the roles of the skull, mandible, mandibular fats, melon, and air sac in binaural receptivity in the horizontal plane (Figure 7). The most distinct feature was found in the comparison between case I and case IV, which led to the conclusion that the amplitude of the received sounds became lower in the forward directions from 76° to 100° after the removal of the mandibular fats, suggesting their important functions to enhance forward sound reception. The average amplitude difference was 1.3 dB, ranging from 0.7 dB to 1.5 dB. The presence of the fatty conduit contributed greatly to shaping the receptivity. The removal of skull led to slight variances between case I and case II at from an incidence angle of 30°. The influences of the mandible, air components, and melon were not noticeable in the horizontal plane at 54 kHz.

**Figure 8:**
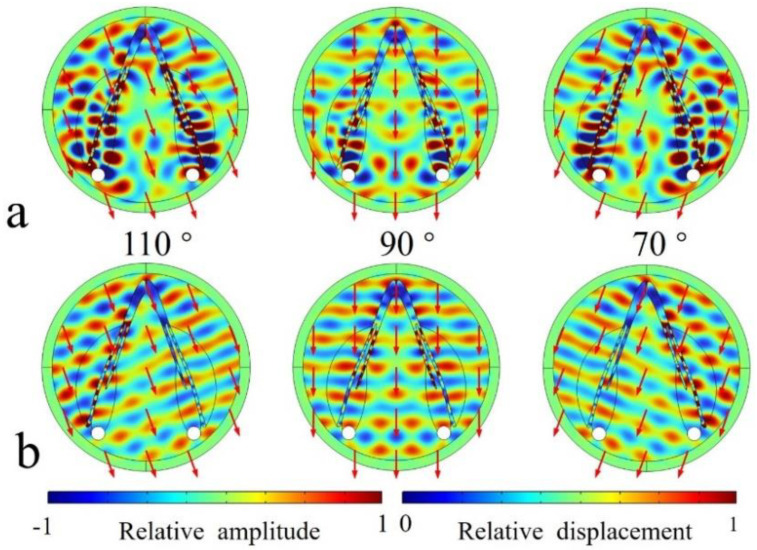
(**a**) The acoustic scattered field of sounds incident from 110°, 90°, and 70° in the horizontal plane examined for the bioinspired model. (**b**) The acoustic scattered field of sounds incident from 110°, 90°, and 70° examined for the bioinspired model in which the silicon was replaced by water.

The sound reception was found to be asymmetrical in the animal model, and using the biomimetic model, we achieved symmetrical sound reception (Figure 8). The wave propagation demonstrated symmetry with reference to the on-axis direction (90°). Sound waves were found to travel bent to the reception sites through the modulation of the low-speed and low-density silicon (Figure 8a). Its replacement by water led to a loss of sound energy travelling to the reception sites (Figure 8b). A large proportion of sound waves after traversing the mandible were not channeled to the reception sites. The comparison of the wave propagation details between these two models suggests the importance of low-speed and low-density silicon in enhancing sound reception.

**Figure 9:**
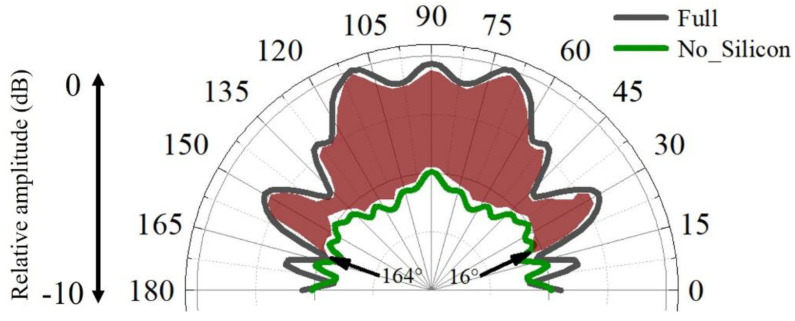
The binaural sound-reception directivities estimated for the full biomimetic model and model in which the low-speed silicon was replaced by water.

The enhancement of the silicon can be further glimpsed in the binaural directivities for the models (Figure 9). The forward reception was efficient from forward incidences, with the highest-sensitivity axis pointing to 110° and 70° and a −3 dB receptivity beam of 88 ranging from 46° to 134°. Without silicon, the amplitude of the received sounds became much lower in the forward directions from 16° to 164°, suggesting their important role in for enhancing forward sound reception. The average amplitude difference was 3.5 dB, ranging from 0.2 dB to 5.9 dB within this angle range.

## 4. Discussion and Conclusions

Sound reception, as one of the two major processes in odontocetes biosonar, has gained much attention ever since the 1960s. Mandibular fats were considered important windows for sounds to enter the head in most papers addressing the sound reception in odontocetes [5,15,17,20]. However, this does not mean the acoustic fats are the only functional structure in sound reception. Mooney et al. found that the finless porpoise had a high sensitivity to acoustic stimuli from the rostral portion of the mandible, and the simulated field in the current paper suggested that the mandible can conduct sounds to the internal mandibular fat and bony ear complex (Figure 5) [24]. The solid mandible can induce vibrations and conduct sounds to the mandibular fats, which can be further carried to stimulate the ear complexes. It contributed to the sound reception as a wave guide [17] and a reverberation structure to improve source localization [18,26,41].

Studies on harbor porpoises, belugas, bottlenose dolphins, and common dolphins have shown that these animals can better sense sounds from forward directions [5,42,43,44,45,46]. The reception directivity at 54 kHz had a high receiving amplitude from forward directions (Figure 7). Future studies are necessary to investigate the frequency response of reception directivity in this species because directional reception has not been examined in the Yangtze finless porpoise, making it difficult to compare simulation and experiment results. Popov et al. found that the monaural directivities had an asymmetrical distribution at low frequencies, depicted by the hearing thresholds at all contralateral sound-source positions, which can also be glimpsed in current study (Figure 6a) [45]. The asymmetry in the binaural reception directivity may also be ascribed to the morphological asymmetry (with the body middle line as reference) in sound-conduction pathways. As stated [45], this phenomenon may be explained by the presence of two or more sound-reception pathways. Aroyan found that in addition to stimulating receptivity peaks on the ipsilateral side as each ear complex, there might be subsidiary pathways for sounds forming receptivity peaks at the ear complex on the opposite side of incidence angles [5]. With such complexity in pathways, the relative weights may respond to frequency and result in variation of the lowest-threshold azimuth, and the whole head may be considered as a volume receptor when considering sound reception from a broadband width.

The simulations and experiments on sound reception in odontocetes have shown that sounds may propagate through many pathways to stimulate the ear complexes [24]. Structures including the skull, mandible, melon, air components, and mandibular fats may were considered to be involved in various ways in conducting sounds, which can be frequency-dependent, direction-dependent, and size-dependent. This paper had limitations in addressing the frequency-related directivities due to the massive computation costs at high frequencies, and the skull reverberation was not examined. Our future work will shed further light on the elastic waves in the skull and mandible as well as their connections to the receptivity, as estimated using the information carried by the ear complex. The mandibular fats were simplified in the model for the reason that the precise boundaries between the mandibular fats and the soft tissues were hard to determine.

Compared to the animal model, we found that the bioinspired and biomimetic model exhibited perfect symmetrical reception. The binaural directivity was apparently symmetrical for the frequency tested, which might be beneficial to acquire interaural differences in amplitude and arrival time of travelling sounds to provide good angular resolution [47]. The symmetry in binaural time or phase difference provides informative cues to determine the difference of the sound’s arrival time at the two ears. The bioinspired design has flexibility, offering the opportunity to modulate reception directivity on demand. The merits of animal sonar can offer insight into developing artificial designs to achieve effective sound reception. Similarly, the low-speed and low-density silicon can greatly improve forward reception, as demonstrated by the red zone in Figure 9. Supposing that the bioreceptor had a reception angle resolution of 5°, The enhancement of the silicon can be over 60 dB, which is calculated by computing (88°/5°) × 3.5 dB. The estimations either in the animal reception model or the biomimetic model, though requiring further examination, at least show that the low impedance was key to enhancing sound reception.

The mandibular fats and the silicon were considered as energy collectors that achieved their role through the lower sound speed and density distributions. Sounds tend to propagate along the low-speed pathway [37,48]. We can use the ray model assumption to track the wave propagation path. The following equation can be used to describe wave propagation in inhomogeneous fluid media:

(2) $$\frac{1}{\lambda (r)}\frac{\partial^{2}p}{\partial t^{2}}=\nabla •\left(\frac{\nabla p}{\rho \left(r\right)}\right)$$

where $\rho \left(r\right)$ and represent density and compressibility coefficients, respectively. Equation (2) can be derived into the following:

(3) $$\frac{d}{ds}\left(n_{B}\frac{dr}{ds}\right)=\nabla n_{B}$$

*s* denotes distance along the ray direction, and the ray curvature vector satisfies the following formula:

(4) K=ds/ds=V/R

where *R* and *V* are the radii of curvature and the unit normal vector of the ray. Accordingly, Equation (4) can be further derived into the following:

(5) $$\left|K\right|=V•\nabla \ln n_{B}$$

where the operator • is the inner product. As $\left|K\right|$ is positive, *V* will direct to the increasing refractive index, corresponding to a low-speed pathway. This basic theoretical analysis explains why the mandibular fats and the silicon can act as an important waveguide to enhance sound reception [48]. We should also note that the there are other substitutes for the low-sound speed and low-density mandibular fats. More importantly, the sound speed and density of the new artificial soft materials are tunable. This flexibility in material properties may provide additional ways to achieve reception performance in demand.

This may help to facilitate artificial reception system designs by concentrating on the key structures, at least at the 54 kHz that we tested here. The biomimetic model did not incorporate structures like melon, skull, and air sacs, but the capability of directional reception was achieved simply by referring to the mandible and acoustic fats, showing the flexibility in biomimetic design. This transition from animal model to biomimetic model can bridge the gap between animal sonar and artificial systems to facilitate sound reception.

## 5. Conclusions

In this paper, we referred to the CT scanning and reconstructions of a Yangtze finless porpoise and ran numerical simulation to examine the sound reception at 54 kHz. The results showed that the mandible and mandibular fats enhanced the sound reception from forward directions in the Yangtze finless porpoise by serving as important energy collectors. The skull showed some modulation on the receptivity by lowering the sounds from front-top directions. The melon and air components had negligible effects on the receptivity at the investigated frequency. The results presented here suggested the potential of referring to the porpoise’s reception system to inspire artificial structures with sound speed and density distributions similar to those of the porpoise. Meanwhile, the bioinspired model has flexibility in adjusting its sound speed and density on demand. These results can enlighten us to design manmade systems to realize spatial selectivity on demand.

## Data Availability

The data that support the findings of this study are available from the corresponding authors upon reasonable request.
